# Exploring Associations Between Self-Compassion, Self-Criticism, Mental Health, and Quality of Life in Adults with Cystic Fibrosis: Informing Future Interventions

**DOI:** 10.1007/s10880-021-09831-y

**Published:** 2021-11-08

**Authors:** S. Kauser, R. Keyte, A. Regan, E. F. Nash, G. Fitch, M. Mantzios, H. Egan

**Affiliations:** 1 Department of Psychology, Faculty of Business, Law and Social Sciences, Birmingham City University, Room C332, The Curzon Building, 4 Cardigan Street, Birmingham, B4 7BD UK; 2 West Midlands Cystic Fibrosis Centre, University Hospitals Birmingham NHS Foundation Trust, Birmingham, UK; 3 North West Midlands Cystic Fibrosis Centre, University Hospitals of North Midlands NHS Trust, Stoke-on-Trent, UK

**Keywords:** Cystic fibrosis, Self-compassion, Quality of life, Mental health, Self-criticism

## Abstract

Self-compassion is increasingly recognised as an important and beneficial factor in quality of life and mental health-related research, but research within the adult cystic fibrosis (CF) population is scarce. In a cross-sectional study, 114 (56 female, 58 male) adults with CF completed and returned a series of validated questionnaires that assessed CF-related quality of life, negative emotional states (depression, anxiety and stress), self-compassion, and self-criticism. Quality of life and self-compassion were positively correlated, and each in turn were inversely correlated with negative emotional states and self-criticism. Negative emotional states correlated positively to self-criticism. Self-compassion and/or self-criticism moderated ten relationships between various sub-domains of quality of life and negative emotions. Psychological interventions that increase self-compassion may be beneficial for enhancing mental health and quality of life for adults with CF.

## Introduction

Cystic fibrosis (CF) is a life-limiting and progressive genetic disorder that affected approximately 10,600 individuals within the United Kingdom (UK) during the year 2021 (Cystic Fibrosis Trust, 2021). Improvements in treatment, including the development of small molecule therapeutics that target the underlying defect, have resulted in many people with CF now surviving into later adulthood years (De Boeck, 2020). Even with these treatments, CF results in recurrent respiratory infections, pancreatic insufficiency, and reduced lung function due to progressive respiratory failure, which is the leading cause of death within the CF population (Goetz & Ren, 2019).

### Mental Health and Quality of Life in CF Populations

Despite improvements in life expectancy, people with CF continue to experience progressively worsening physical and psychological symptoms (Cronly et al., 2019a). Psychological comorbidities of depression and anxiety are extremely prevalent across the CF population (Quittner et al., 2014). Several studies have shown that mental health variables such as stress, anxiety, and depression are negatively linked with quality of life (Cronly et al., 2019a; Havermans et al., 2008; Platten et al., 2013; Riekert et al., 2007; Yohannes et al., 2012). Using the Cystic Fibrosis Quality of Life Questionnaire (CFQoL), anxiety and depression significantly correlated with physical functioning, social functioning, treatment issues, chest symptoms, emotional functioning, concerns for the future, interpersonal relationships, body image and career concerns (Yohannes et al., 2012).

In people with CF, poor mental health is more strongly associated than poor physical health with lower quality of life, highlighting the importance of treating mental health to maintain quality of life. This is particularly important as physical challenges increase with age (Cronly et al., 2019a), and adults with CF may engage in health risk behaviours as a way of coping with their disease (Keyte et al., 2019a, b, 2020). The associations between positive mental health, better physical health, and higher health-related quality of life have been clearly demonstrated (Cronly et al., 2019b). With a projected 75% increase of the CF adult population by 2025 (Burgel et al., 2015), increased efforts to support both psychological and physical health needs in the later adulthood years are required.

### Self-Compassion

Self-compassion is an important and beneficial resource for supporting mental health and quality of life for adults with CF. Self-compassion is defined as the act of being open to one’s own suffering by implementing a kind, understanding and non-judgemental approach towards challenges (Neff, 2003a, b). Neff specified three positive and core elements of self-compassion, which include self-kindness, common humanity, and mindfulness, and three negative elements including self-judgement, isolation, and over-identification. In both clinical and non-clinical populations, higher self-compassion levels have been associated with better ability to cope with pain, increased well-being in later adulthood years, adaptive responses to illness, and increased engagement in health-promoting behaviours (Allen et al., 2012; Egan & Mantzios, 2018; Egan et al., 2019; Mantzios & Egan, 2017; Sirois et al., 2015). Research in self-compassion and mental health over the past decade indicated a large effect size for the relationship between both concepts, linking increased levels of self-compassion with fewer symptoms of depression, anxiety, and stress amongst non-CF populations (MacBeth & Gumley, 2012). Self-criticism, which can be conceptualised as opposite to self-compassion, is described as engaging in a constant and harsh evaluation of oneself (Blatt & Zuroff, 1992). Self-criticism has been negatively associated with quality of life and positively associated with depression, anxiety, and stress (Iancu et al., 2015; Pinto‐Gouveia et al., 2014; Priel & Shahar, 2000; Zhang et al., 2019). Research in both self-compassion and self-criticism may offer an insight as to which psychological interventions are most valuable for people with CF.

Psychological interventions and therapies that draw upon the foundations of self-compassion, including loving-kindness meditation (LKM), compassion-focussed therapy (CFT), and mindful self-compassion (MSC) training, have been successful in promoting positive health outcomes (Gilbert, 2009; Ilies et al., 2019; Neff & Germer, 2013; Salzberg, 1995). The theoretical connectedness between mindfulness and self-compassion suggests that both concepts provide key elements to these interventions. MacBeth and Gumley (2012) highlighted the use of compassion-based therapies to reduce psychological distress through the acquisition of an open and compassionate attitude, whereby the individual’s relationship with their challenges are reformed, resulting in the development of a healthier relationship with oneself. Previous literature demonstrated that self-compassion and mindfulness-based interventions are beneficial in reducing anxiety, depression, and stress symptoms, decreasing self-criticism and improving quality of life (Chiesa & Serretti, 2009; Evans et al., 2008; Godfrin & Van Heeringen, 2010; Segal et al., 2002; Shahar et al., 2015).

### Self-Compassion: A Construct Relevant to Quality of Life and Mental Health in Adults with CF

Prior work on self-compassion in non-CF populations suggests that self-compassion could help people cope with the multiple challenges of living with CF that have been shown to affect quality of life, including physical health, body image, treatment burden, future concerns, and employment (Brion et al., 2014; Herriot & Wrosch, 2020; Phillips, 2018; Seekis et al., 2017; Rajabi & Ghezelsefloo, 2020). Individuals with CF face extensive physical health challenges and symptomatology including coughing, breathlessness, and pain (Stenekes et al., 2009). People with CF often experience pulmonary exacerbations, characterised by changes in cough, sputum production, dyspnoea, and decreases in energy level, appetite, and weight loss, resulting in increased emotional distress (de Boer et al., 2011; Goss & Burns, 2007; Schmid‐Mohler et al., 2019). One recent study showed that self-compassion predicted lower levels of daily physical symptoms as well as fewer rises in chronic illness amongst people in advanced old age (Herriot & Wrosch, 2020), suggesting that self-compassion may be a useful tool in supporting physical health in adults with CF particularly when approaching end of life.

Body image perception is relevant within the CF literature as weight maintenance can present continuous difficulties for people with CF, negatively affecting psycho-social functioning (Egan et al., 2020). A critical aspect of CF patient care involves the monitoring of body mass index (BMI). Patients with CF who received enteral tube feeding reported lower self-esteem, a poorer quality of life, and less satisfaction with their body image (Abbott et al., 2007). In a non-CF population, low levels of self-compassion have been found to mediate the relationship between body image disturbance and psychological distress (Przezdziecki et al., 2013). Similarly, a study exploring the effectiveness of a self-compassion writing task revealed the self-compassion writing group showed higher post-treatment body appreciation when compared to self-esteem and control groups (Seekis et al., 2017). The findings by Przezdziecki et al. (2013) and Seekis et al. (2017) provide promising evidence for the role of self-compassion for people at risk of experiencing body image disturbance.

CF treatment regimens are extremely burdensome (Knudsen et al., 2016) with detrimental impacts on both quality of life and psychological well-being (Sawicki & Goss, 2015), and low adherence is a major concern (Keyte et al., 2017). The benefits of self-compassion have not been thoroughly explored within the CF population for possible positive associations with treatment-related challenges. However, in a sample of patients diagnosed with HIV, results highlighted associations between self-compassion and better medical and treatment adherence, as well as lower levels of stress and anxiety (Brion et al., 2014). Therefore, understanding more about self-compassion in CF may prove beneficial for this population.

Having CF understandably raises concerns for the future, and people with high levels of concerns have increased levels of anxiety and stress (Yohannes et al., 2012). People with higher self-compassion have a more positive future outlook (Phillips, 2018) and use more adaptive coping mechanisms such as cognitive restructuring when considering future suffering (Allen & Leary, 2010). Challenges in employment and financial insecurity for people with CF add to concerns for the future (Havermans et al., 2009); having higher self-compassion can reduce job stress and increase job-related well-being (Rajabi & Ghezelsefloo, 2020). Self-compassion interventions may therefore be valuable for increasing employment-related affective well-being as well as reducing employment-related stress in adults with CF.

### Rationale, Aims, and Hypothesis

Self-compassion is increasingly recognised as an important factor in quality of life and mental health research. It is therefore important that we understand more about how self-compassion could be integrated into both clinical care and self-care within the adult CF population. It is possible that any benefits of mindfulness for people with CF may also be observed in self-compassion and compassion-based interventions (Egan & Mantzios, 2016; Mantzios & Egan, 2016; Mantzios et al., 2016). Thus, the current research aimed to firstly explore the relationships between quality of life, negative emotional states (depression, anxiety, and stress), self-compassion, and self-criticism and secondly to explore the moderating effects of self-compassion and self-criticism on the relationship between quality of life and negative emotional states within an adult CF population.

We predicted that a negative correlation would be observed between negative emotional states and quality of life. We also predicted that self‐compassion would be associated with increased quality of life and decreased negative emotional states, and that self-criticism would result in correlations in the opposite direction. Additionally, we hypothesised that self-compassion would moderate the relationship between negative emotions and quality of life. Similarly, we hypothesised that self-criticism would moderate relationships between negative emotional states and quality of life.

## Method

### Participants

We recruited 160 English-speaking adults living in the UK who had a confirmed diagnosis of CF. Of these, 114 participants (71% response rate) completed and returned the questionnaires. Participant demographic and medical data are presented in Table 1. Recruitment took place at two regional adult CF Centres in the Midlands over a period of 8 months on pre-arranged days for data collection, using a purposeful sampling method. Participant invitations to take part in the study occurred during a routine outpatient appointment or after inpatient admission to the ward. Ethical approval was obtained by the Universities Ethical Committee, the Health Research Authority (HRA) via an NRES Committee (REC reference: 19/NE/0183), and the R&D departments at each research site.

**Table 1:** Participant demographic and medical data

Variable	Participants (*n* = 114)
Gender	
Male	58
Female	56
Age (years)	
Range	18–70
Mean (SD)	32.36 (11.50)
Ethnicity	
White	100
Asian or Asian British	8
Mixed	5
Cypriot	1
Highest level of education	
University degree	52
A levels or BTEC	41
GCSE	18
Not applicable	3
Current employment status	
Employed	65
Self-employed	12
Un-employed	28
Retired	9
BMI	
Range	16.75–36.70
Mean (SD)	23.47 (3.82)
FEV_1_ (%)	
Range	20–115
Mean (SD)	62.70 (22.65)
FVC (%)	
Range	37–123
Mean (SD)	79.29 (19.65)

### Materials

#### Demographic Information Form

Participants reported their date of birth, gender, ethnicity, highest level of education, and employment status.

#### Medical Information Form

We collected the following information from participants’ medical records: forced expiratory volume in 1 second (FEV_1_), forced vital capacity (FVC) and BMI.

#### Self-Compassion Scale (SCS; Neff, 2003a)

The SCS is a 26-item measure composed of six subscales: self-kindness, self-judgement, common humanity, isolation, mindfulness, and over-identification. Possible responses range from 1 (*almost never*) to 5 (*almost always*). Total scores were computed following the calculation of a total mean, as recommended by Neff (2003a). Higher scores indicate a greater amount of self-compassion. Sample items include “I’m kind to myself when I’m experiencing suffering” (subscale: self-kindness) and “when I fail at something important to me I become consumed by feelings of inadequacy” (subscale: over-identification). The SCS demonstrated good internal consistency, test–retest reliability, and convergent validity in clinical populations (Castilho et al., 2015). In the present sample, Cronbach’s alpha for the total score was .94.

#### The Functions of Self-Criticizing/Attacking Scale (FSCS; Gilbert et al., 2004)

The FSCS is a 21-item measure composed of two subscales: self-correction and self-persecution. Possible responses range from 0 (*not at all like me*) to 4 (*extremely like me*). Higher scores indicate a greater amount of self-criticism. Sample items include “I get critical and angry with myself to keep myself in check” (subscale: self-correction) and “I get critical and angry with myself because, if I punish myself I feel better” (subscale: self-persecution). Gilbert et al. (2004) conducted a principal components analysis and reported good internal consistency for the FSCS scale components. In the present sample, Cronbach’s alpha was .89.

#### The Depression Anxiety Stress Scale (DASS; Lovibond & Lovibond, 1995)

The DASS is a 21-item measure composed of three subscales: depression, anxiety, and stress. Possible responses range from 0 (*did not apply to me at all*) to 3 (*applied to me very much, or most of the time*). Total scores were computed following the scores multiplied by two, as recommended by Lovibond and Lovibond (1995). Higher scores indicate a greater severity of negative emotional states of depression, anxiety, and stress. Sample items include “I felt that life was meaningless” (subscale: depression) and “I felt scared without any good reason” (subscale: anxiety). The DASS demonstrated internal consistency and concurrent validity in the acceptable to excellent ranges in clinical populations (Antony et al., 1998). In the present sample, Cronbach's alpha for the total score was .95.

#### CFQoL; Gee et al. (2000)

The CFQoL is a 52-item measure composed of nine subscales: physical functioning (1), social functioning (2), treatment issues (3), chest symptoms (4), emotional functioning (5), concerns for the future (6), interpersonal relationships (7), body image (8) and career concerns (9). Possible responses for scales one to four range from 1 (*all of the time*) to 6 (*never*), whilst scales five to nine range from 1 (*strongly agree*) to 6 (*strongly disagree*). Higher scores indicate a greater level of quality of life. Sample items include “The way that my CF has made me look because of my height/weight makes life less enjoyable” (subscale: body image) and “During the last two weeks, my CF has made me feel lacking in energy” (subscale: physical functioning). The CFQoL showed robust internal reliability, concurrent validity and test–retest reliability in a clinical CF population (Gee et al., 2000). In the present sample, Cronbach’s alpha for the overall score was .97.

### Procedure

Following the “consent to contact” ethical procedure, a clinician at the CF centre introduced the research study to the potential participant. If agreed, the first author then provided potential participants with a verbal overview of what the study entailed, followed by a participant information form. Once verbally agreed, participants provided written informed consent and then complete the demographics form and the questionnaires. On average, the questionnaires took 20 minutes to complete. The first author collected data from participants’ medical records (FEV_1_, FVC, BMI). Following completion, the first author issued participants with a debrief form with an overview of the study and researchers contact details should participants decide to withdraw their data later, though none did so.

### Analyses

We used IBM SPSS v25 to analyse the data. Preliminary analyses examined for outliers and met assumptions of normality and linearity (Field, 2013). We computed bivariate correlations to explore associations between self-compassion, self-criticism, negative emotional states, and quality of life. Moderation analyses further examined whether self-compassion or self-criticism moderated the relationship between negative emotional states and quality of life total scores and subscale scores. We used PROCESS v3.5 (Model 1) to interpret moderation effects which included a bootstrap sample of 10,000, and variables centred to their means (Hayes, 2017). Calculations of simple effects coefficients for three values of the moderator included: 1 SD below the mean, at the mean, and 1 SD above the mean. Bias-corrected confidence intervals (CI) and the bootstrapping procedure were used to attribute statistical significance of the moderator. To reduce Type I error, the research team applied a Bonferroni correction procedure for multiple hypothesis testing using a correction applied to the alpha level (Field, 2013). The alpha level (0.05) was divided by the number of tests conducted within self-compassion and self-criticism moderation analyses.

## Results

Pearson correlation coefficients between quality of life (CFQoL), negative emotional states (DASS), self-compassion (SCS), and self-criticism (FSCS) total scores are presented in Table 2.

**Table 2:** Bivariate correlations between quality of life, negative emotional states, self-compassion, and self-criticism total scores

	1	2	3	4
1. CFQoL	–			
2. DASS	− .639**	–		
3. SCS	.526**	− .697**	–	
4. FSCS	− .552**	.575**	− .636**	–

*CFQoL* Cystic Fibrosis Quality of Life Scale, *DASS* Depression Anxiety Stress Scale, *SCS* Self-Compassion Scale, *FSCS* Functions of Self-Criticizing/Attacking Scale

**Correlation is significant at the .01 level

Pearson correlation coefficients between quality of life, negative emotional states, self-compassion and self-criticism subscale scores are presented in Table 3. We found negative associations between all nine CFQoL subscales and all three DASS subscales (depression, anxiety, and stress).

**Table 3:** Bivariate correlations between quality of life, negative emotional states, self-compassion and self-criticism subscale scores

	1	2	3	4	5	6	7	8	9	10	11	12	13	14	15	16	17	18	19	20
1. CFQoL—physical functioning^a^	–																			
2. CFQoL—social functioning^a^	.788**	–																		
3. CFQoL—treatment issues^a^	.567**	.537**	–																	
4. CFQoL—chest symptoms^a^	.697**	.475**	.458**	–																
5. CFQoL—emotional functioning^a^	.679**	.679**	.516**	.658**	–															
6. CFQoL—concerns for the future^a^	.425**	.373**	.395**	.478**	.492**	–														
7. CFQoL—interpersonal relationships^a^	.550**	.585**	.503**	.429**	.625**	.517**	–													
8. CFQoL—body image^a^	.207*	.293**	.189*	.122	.384**	.210*	.429**	–												
9. CFQoL—career concerns^a^	.450**	.473**	.498**	.278**	.482**	.344**	.567**	.400**	–											
10. DASS—stress^b^	− .465**	− .556**	− .303**	− .270**	− .695**	− .357**	− .565**	− .347**	− .370**	–										
11. DASS—anxiety^b^	− .500**	− .527**	− .297**	− .384**	− .636**	− .399**	− .547**	− .331**	− .314**	.756**	–									
12. DASS—depression^b^	− 491**	− .504**	− .336**	− .366**	− .710**	− .399**	− .618**	− .335**	− .408**	.813**	.718**	–								
13. SCS—self-kindness^c^	.181	.312**	.167	.132	.441**	.127	.329**	.237*	.192*	− .501**	− .365**	− .468**	–							
14. SCS—self-judgement^c^	− .394**	− .472**	− .314**	− .384**	− .695**	− .440**	− .584**	− .341**	− .399**	.658**	.520**	.613**	− .579**	–						
15. SCS—common humanity^c^	− .017	.074	.032	− .058	.156	.074	.151	.024	.002	− .275**	− .152	− .234*	.597**	− .227*	–					
16. SCS—isolation^c^	− .308**	− .456**	− .272**	− .319**	− .586**	− .453**	− .618**	− .341**	− .360**	.642**	.484**	.642**	− .375**	.702**	− .178	–				
17. SCS—mindfulness^c^	.059	.203*	.107	.005	.275**	.167	.208*	.138	.120	− .395**	− .245**	− .403**	.712**	− .375**	.661**	− .355**	–			
18. SCS—over-identification^c^	− .290**	− .411**	− .230*	− .253**	− .584**	− .409**	− .506**	− .318**	− .389**	.695**	.486**	.650**	− .424**	.757**	− .276**	.797**	− .427**	–		
19. FSCS—self-correction^d^	− .263**	− .450**	− .266**	− .282**	− .526**	− .423**	− .438**	− .179	− .263**	.564**	.398**	.482**	− .358**	.559**	− .134	.525**	− .281**	.576**	–	
20. FSCS—self-persecution^d^	− .330**	− .428**	− .264**	− .281**	− .535**	− .422**	− .442**	− .331**	− .267**	.489**	.401**	.530**	− .356**	.582**	− .250**	.509**	− .365**	.542**	.568**	–

*Correlation is significant at the .05 level

**Correlation is significant at the .01 level

^a^Subscales of the Cystic Fibrosis Quality of Life Scale (CFQoL)

^b^Subscales of the Depression Anxiety Stress Scale (DASS)

^c^Subscales of the Self-Compassion Scale (SCS)

^d^Subscales of the Functions of Self-Criticizing/Attacking Scale (FSCS)

Further analyses of moderation effects explored self-compassion and self-criticism as potential moderators of observed relationships between quality of life and negative emotional states (see Table 4).

**Table 4:** Conditional effects of self-compassion and self-criticism scores on relationships between quality of life and negative emotional state domains

Relationship	Moderator		*β*	*p*	95% CI	
CFQoL-body image and DASS-anxiety	SCS	− 1 *SD*	− .046	.378	− .150	.057
		At the mean	− .116	.011	− .205	− .027
		+ 1 *SD*	− .185	.008	− .322	− .049
CFQoL-treatment issues and DASS-anxiety	SCS	− 1 *SD*	− .012	.809	− .114	.089
		At the mean	− .099	.009	− .174	− .025
		+ 1 *SD*	− .186	.002	− .305	− .068
CFQoL-career issues and DASS-stress	FSCS	− 1 *SD*	− .174	.000	− .267	− .080
		At the mean	− .094	.007	− .160	− .027
		+ 1 *SD*	− .014	.792	− .115	.088

*CFQoL* Cystic Fibrosis Quality of Life Scale, *DASS* Depression Anxiety Stress Scale, *SCS* Self-Compassion Scale (total score), *FSCS* Functions of Self-Criticizing/Attacking Scale (total score)

**Fig. 1:**
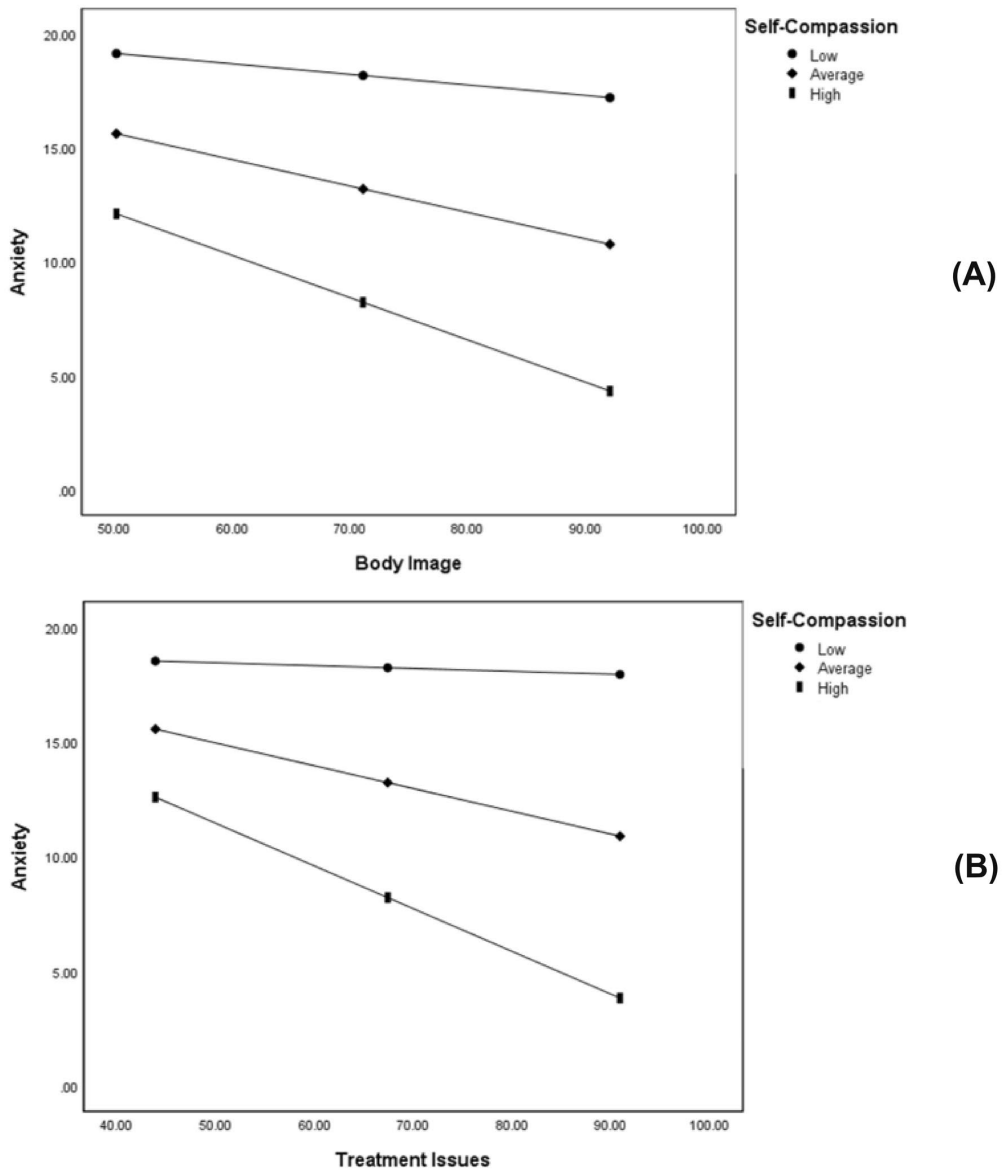
Self-Compassion as a moderator of the relationship between DASS-anxiety scores and quality of life domain scores (A = Body Image, B = Treatment Issues). Lines depict modelled relationships at different levels of the Self-Compassion Scale (total score): 1 *SD* below the mean of SCS (Low), mean SCS (Average), and 1 *SD* above the mean of SCS (High)

Average and high levels of self-compassion significantly and negatively moderated the relationship between body image and anxiety. People who felt more positive about their body image had lower levels of anxiety when self-compassion moderated the relationship at average and high levels (see Table 4 and Fig. 1).

Average and high levels of self-compassion also significantly and negatively moderated the relationship between treatment issues and anxiety. People who reported fewer treatment-related issues had lower levels of anxiety when self-compassion moderated the relationship at average and high levels (see Table 4 and Fig. 1).

**Fig. 2:**
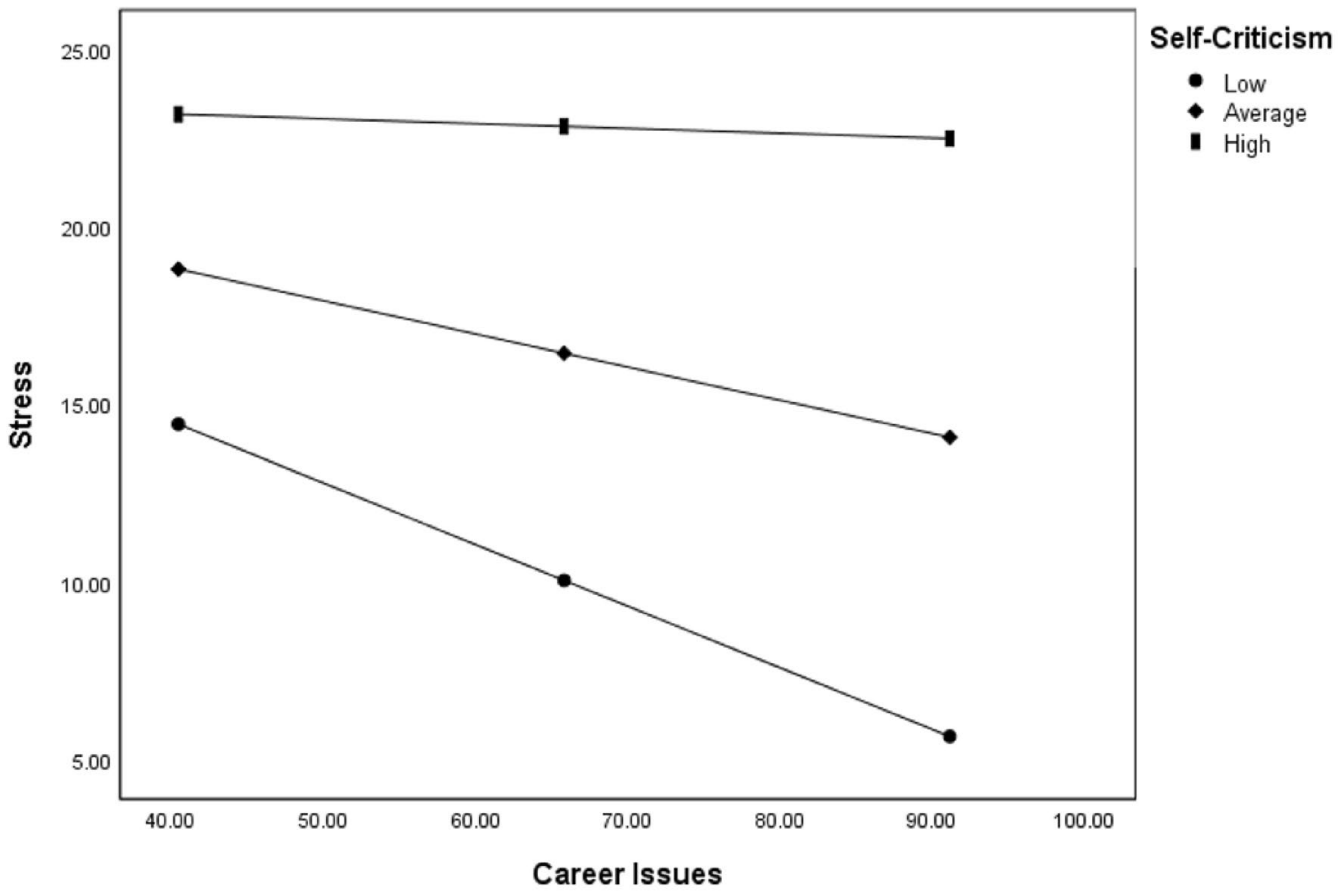
Self-Compassion as a moderator of the relationship between DASS-stress scores and quality of life domain scores. Lines depict modelled relationships at different levels of the Self-Compassion Scale (total score): 1 *SD* below the mean of SCS (Low), mean SCS (Average), and 1 *SD* above the mean of SCS (High)

Low and average levels of self-criticism significantly and negatively moderated the relationship between career issues and stress. Participants who reported more positive career issues had lower levels of stress when self-criticism moderated the relationship at low and average levels (see Table 4 and Fig. 2).

Using a Bonferroni adjusted alpha level of 0.025 for self-compassion moderations, the relationships between body image and anxiety (*p* = .011) and treatment issues and anxiety (*p* = .009) remained significant. However, when using a Bonferroni adjusted alpha level of 0.017 for self-criticism moderations, only the relationship between career issues and stress (*p* = .007) remained significant. Both relationships between treatment issues and anxiety (*p* = .026), and chest symptoms and anxiety (*p* = .076) became insignificant after the Bonferroni correction.

Moderation analyses for self-compassion and self-criticism subscales are available from the author on request.

## Discussion

We aimed to explore relationships between quality of life, negative emotional states, self-compassion, and self-criticism, including the moderating role of self-compassion and self-criticism within an adult CF population. Individuals who endorsed better quality of life tended to report greater self-compassion, whereas those who reported more severe negative emotional states endorsed greater self-criticism. Further investigation of moderation effects suggested that self-compassion or self-criticism moderated three relationships between quality of life and negative emotional states.

Our findings on the relationships between quality of life and mental health align with previous findings in this population (Cronly et al., 2019a; Havermans et al., 2008; Platten et al., 2013; Riekert et al., 2007; Yohannes et al., 2012). Literature on self-compassion and self-criticism within the adult CF population has been largely unexplored. The present results demonstrate positive associations between increased self-compassion, negative emotional states, and enhanced levels of quality of life in adults with CF, which corroborates with prior self-compassion studies in non-CF populations (MacBeth & Gumley, 2012; Pinto‐Gouveia et al., 2014). Both the current study and previous findings report associations between increased levels of self-criticism with higher levels of negative emotional states and quality of life (Pinto‐Gouveia et al., 2014; Iancu et al., 2015).

### Self-Compassion as a Moderator of the Relationship Between Quality of Life Domains and Anxiety

The present study suggests that self-compassion may moderate the association of body image concerns with anxiety in people with CF. As body image perception related to weight maintenance and tube feeding negatively affects psycho-social functioning for adults with CF (Abbott et al., 2007; Egan et al., 2020), promoting accessible and effective ways to increase self-compassion could prove beneficial. Psychological practices such as loving-kindness meditation, a self-care technique to boost well-being and self-acceptance, and compassion-focussed therapy centring on expanding self‐compassionate qualities and skills may be helpful for people who report lower quality of life relating to body image concerns.

We also found that self-compassion also moderated the relationship between treatment issues and anxiety. Both the current study and previous literature demonstrate the impact of CF treatment burden upon psychological well-being (Sawicki & Goss, 2015). Self-compassionate people recognise that experiencing life difficulties is inevitable and accept this reality with self-kindness rather than self-judgement when confronted with painful experiences (Neff, 2003a). Thus, self-compassion may be an important resource for adults with CF when experiencing challenging periods in life (for example, during a health or lung function decline which can exacerbate treatment issues). Treatment regimens form a significant part of daily life for people with CF (Knudsen et al., 2016). Focussing on increasing levels of self-compassion with mindful self-compassion training could reduce the psychological impact of changes in health-related quality of life.

### Limitations and Future Research

We identified three limitations within this study. The first limitation is that the study did not account for whether or not individuals had ever engaged in any self-compassion or mindfulness-based interventions. The second limitation involved a lack of control for mental health diagnosis and medication use. Addressing these two limitations would have allowed for stronger conclusions to be drawn. Thirdly, the study presents cross-sectional data which, while a necessary first step in identifying relationships between the variables, makes it difficult for causal associations to be interpreted. Future research should therefore explore the effects of self-compassion on quality of life and mental health in adults with CF through psychological interventions which draw upon self-compassion and mindfulness-based concepts. Given the physical respiratory difficulties faced by individuals with CF, researchers should consider the type of intervention administered to facilitate the needs of this population. For example, practises that rely heavily upon focussed breathing may prove particularly difficult for individuals with CF.

### Conclusion

We found that quality of life, negative emotional states, self-compassion, and self-criticism were all significantly associated, and that self-compassion and self-criticism moderate the relationship between negative emotional states subscales and five quality of life subscales. Understanding the elements of self-compassion and mindfulness in adults with CF may be useful in developing future interventions that will continue to support the psychological and physical health needs of this population. The current findings suggest that psychological interventions which increase self-compassion could be beneficial for enhancing better mental health and quality of life for adults with CF.

## Data Availability

The data that support the findings of this study are available on request from the corresponding author. The data are not publicly available due to public availability violating the consent that was given by research participants.

## Declarations

### Research Involving Human and Animal Participants

This manuscript does not contain any studies with animals.
